# Multi-task learning for predicting SARS-CoV-2 antibody escape

**DOI:** 10.3389/fgene.2022.886649

**Published:** 2022-08-11

**Authors:** Barak Gross, Roded Sharan

**Affiliations:** School of Computer Science, Tel Aviv University, Tel Aviv, Israel

**Keywords:** multi-task learning, neural network, escape prediction, coronavirus, receptor binding domain

## Abstract

The coronavirus pandemic has revolutionized our world, with vaccination proving to be a key tool in fighting the disease. However, a major threat to this line of attack are variants that can evade the vaccine. Thus, a fundamental problem of growing importance is the identification of mutations of concern with high escape probability. In this paper we develop a computational framework that harnesses systematic mutation screens in the receptor binding domain of the viral Spike protein for escape prediction. The framework analyzes data on escape from multiple antibodies simultaneously, creating a latent representation of mutations that is shown to be effective in predicting escape and binding properties of the virus. We use this representation to validate the escape potential of current SARS-CoV-2 variants.

## 1 Introduction

Since 2019, severe acute respiratory syndrome coronavirus 2 (SARS-CoV-2), accounted for more than 500 million infections and more than six million deaths worldwide according to World Health Organization (WHO, 2022). Though the virus mutates rapidly, only a small minority of mutations are expected to impact the virus phenotype and increase its fitness advantage. Such mutations might alter properties of the virus such as: pathogenicity, infectivity, transmissibility and/or antigenicity. Due to the virus’ high infectivity and rapid mutability, in early stages of the pandemic such mutations of concern started to appear. For example, D614G was noted to be increasing in frequency in April 2020 and to have emerged independently several times in the global SARS-CoV-2 population. Subsequent studies indicated that D614G confers a moderate advantage for infectivity (Hou et al., 2020; Yurkovetskiy et al., 2020) and transmissibility (Volz et al., 2022).

Although several antibodies and vaccines showed good clinical results, recognizing mutations that impact the escape from antibodies and vaccines is still a major question in the battle against SARS-CoV2. The receptor binding domain (RBD) region is a sub-region of the SARS-CoV-2 spike glycoprotein that mediates viral attachment to ACE2 receptors. The RBD is a major determinant of host range and a dominant target of neutralizing antibodies, promoting systematic studies of mutations to the RBD region and their impact on a variety of attributes including binding (Starr et al., 2020), antibody escape (Starr et al., 2021a; Greaney et al., 2021b; Starr et al., 2021b) and more. There are fewer studies that consider multiple mutations (Li et al., 2020; Barton et al., 2021), covering only a handful of them due to the infeasible number of experiments needed to iterate over all possibilities.

The on-going emergence of variants with dozens of mutations motivates computational approaches to study the effect of multiple mutations. These include structural-based approaches (Rienzo et al., 2021; Bozdaganyan et al., 2022) or approaches that directly use deep mutational scans. An example is the escape calculator (Greaney et al., 2021a) which aggregates data about antibody escape using an interpolation based approach, thus allowing for a quantitative scoring of the antigenic effects of arbitrary combinations of mutations. Deep learning methods (Goodfellow et al., 2016) have become the method of choice for diverse data science applications including the analysis of coronavirus data. Specifically, Hie et al. (2021) applied a masked language model approach to a data set of more than 1 million SARS-CoV-2 sequences. Using the language model they ranked mutations based on semantic change (distance between wildtype and mutated sequence) and grammaticality (probability for mutation under the resulting model), thus aiding in identification of mutations that evade the immune system. But they did not address any antibodies or vaccines in their work.

In this paper, we try to combine the best of both worlds–aggregating escape data based on experimental data a la (Greaney et al., 2021a), while using deep learning methods, like in (Hie et al., 2021)—to tackle the challenge of predicting antibody escape potential. Our approach uses the paradigm of multi-task learning, where multiple learning tasks are solved at the same time in order to exploit commonalities and differences across tasks. We show that using a multi-task approach to learn escape data endows us with a representation that can be useful in multiple prediction scenarios. We further apply our approach to analyze the common variants of concern.

## 2 Results

We developed a framework to assess the effect of mutations in the RBD on viral escape, both with a single-task approach and a multi-task approach. We tested our framework using experimental antibody escape data and compared the multi-task and single-task approaches. We demonstrate that multi-task learning helps reduce variance and improve performance. Moreover, we show that using multi-task learning yields an informative representation of the RBD sequence that can be subsequently used to predict multiple properties.

### 2.1 Multi-task learning improves antibody escape recognition

Our main training data set is taken from Greaney et al. (2021b) and contains systematic single amino-acid substitutions in the RBD region and their effects on escape probability with respect to each one of several antibodies. From the aforementioned data two tasks were derived: classifying a mutation as significant for escape and predicting (regressing) its escape probability. To this end, we developed neural network models that either consider one antibody at a time (single-task) or multiple antibodies simultaneously (multi-task). The architectures and training process of these models are detailed in the Materials and Methods.

**FIGURE 1:**
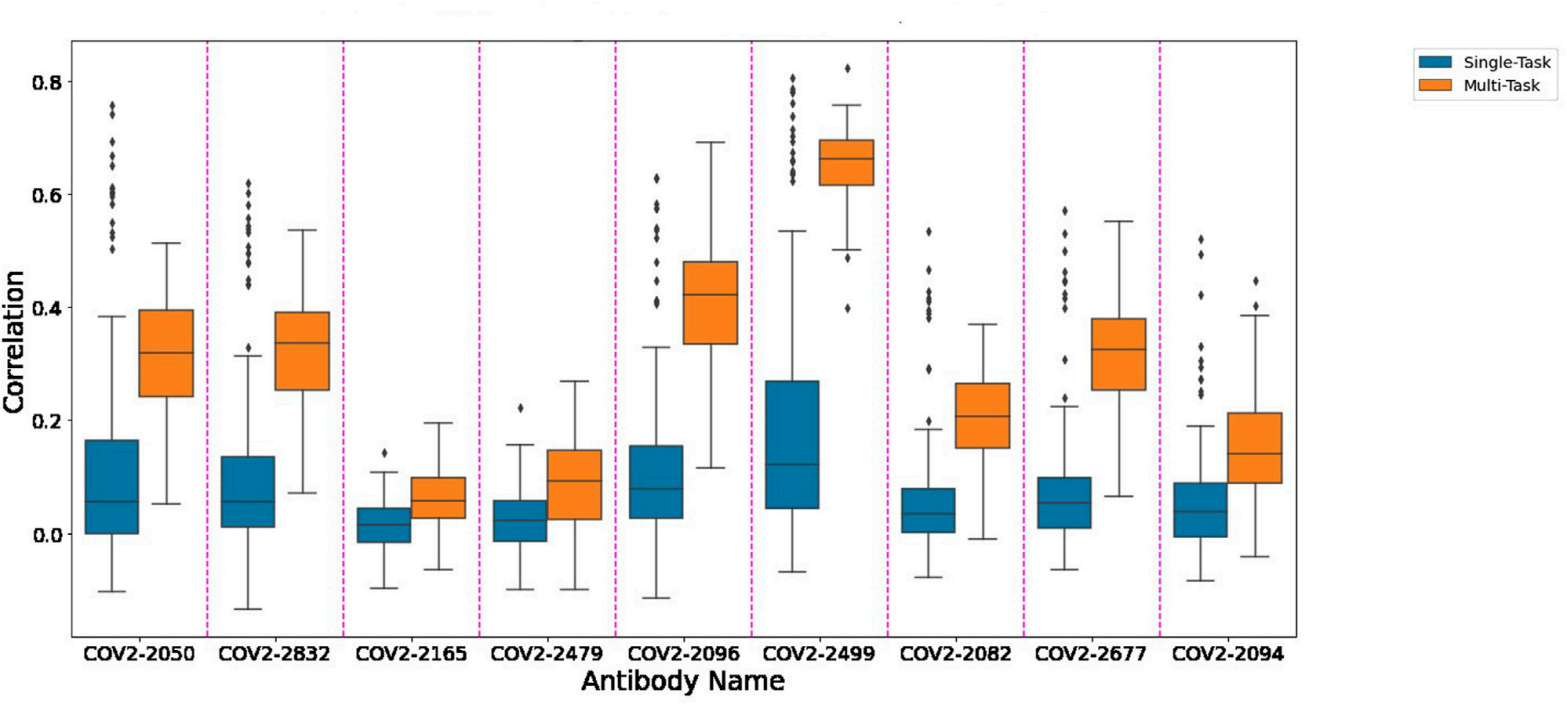
A comparison of single-task and multi-task performance in predicting escape probability.

**FIGURE 2:**
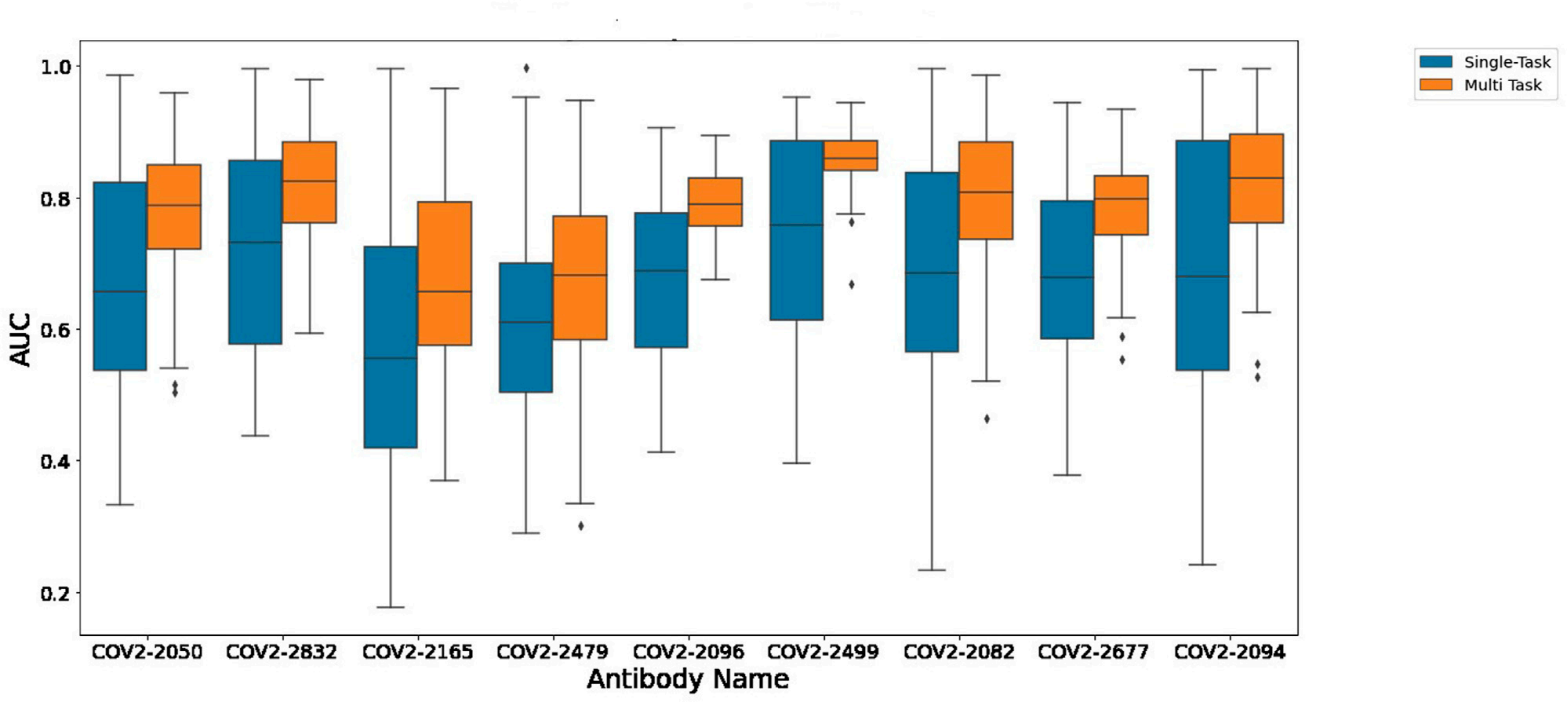
A comparison of single-task and multi-task performance in binary escape prediction.

Figure 1 depicts the (distribution of) Pearson correlation between predicted and measured escape probabilities across 9 antibodies, comparing between the single-task and multi-task approaches. Similarly, Figure 2 depicts the area under the ROC curve for the corresponding classification task. It is evident that the multi-task approach reduces variance and increases mean performance for both regression and classification tasks, respectively.

### 2.2 Analysis of the induced embedding

**FIGURE 3:**
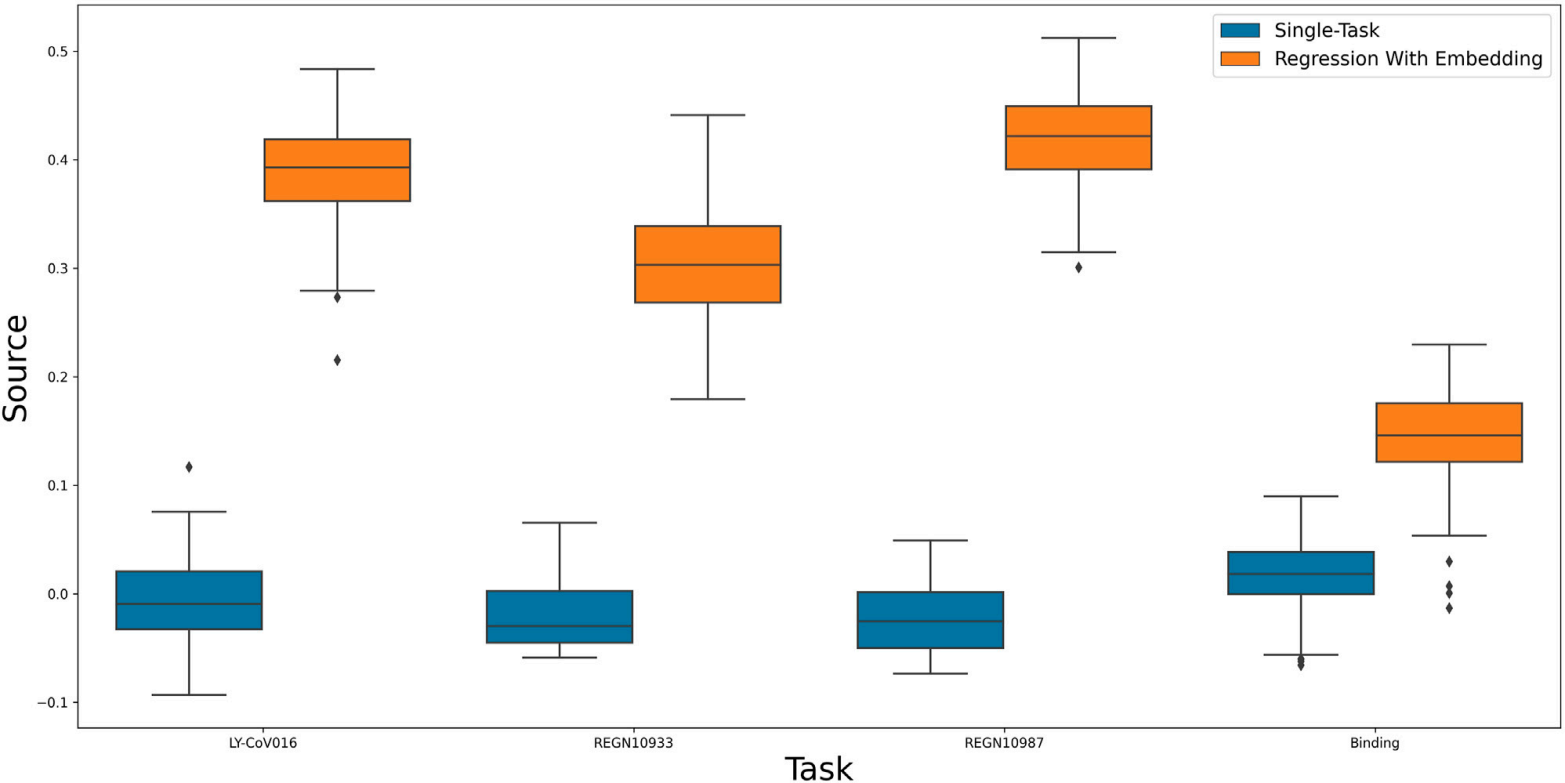
A comparison of a single-task neural network to linear regression of multi-task induced embedding.

After establishing the utility of our predictive model, We aim to further use it to find an informative representation of mutations that is more compact than the sequence of amino acids, while also preserving antibody escape information. Such a representation will allow us to test the predictive power of our model with respect to yet unseen properties. As a first test, we calculate viral escape of single amino-acid substitution from new, yet unseen antibodies: LY-CoV016, REGN10987 and REGN10933 Starr et al. (2021b). Figure 3 shows that the embedding-based predictions outperform the original neural network. This result indicates the power of the latent representation compared to the original amino-acid sequence.

As a second test, we checked the utility of the representation in predicting the effect of a single amino-acid substitution on the binding of the spike protein to ACE2. Specifically, binding affinity is given as the difference between the log of the dissociation constant of the mutation with respect to wildtype. As the binding is vital for viral entry, we assumed the learned representation could encompass useful information about it. Figure 3 confirms this assumption and shows that using the learned representation leads to improved predictions. In conclusion, the embedding was able to encode useful data regarding sites and mutations and apply them to new tasks successfully.

## 3 Materials and methods

### 3.1 Data representation

Greaney et al. compiled a data set containing the escape information of about 2,000 single amino-acid substitutions in the RBD with respect to nine monoclonal antibodies Greaney et al. (2021b). Since the amino-acid changes are all in the RBD region, we can treat our input as a subsequence of the original Spike protein, reducing the representation to a 201-long character string.

The original escape information is given as probabilities. In order to create the viral-escape classification task from the continuous data, we followed Starr et al. Starr et al. (2021b) and chose 10 times the median escape across all sites as threshold for significant escape for each antibody and gave a label of 1 to samples that exceeded the threshold.

### 3.2 Neural network architecture and training

We use a neural network that receives as an input a string of fixed length *n* = 201 over an alphabet of size *m* = 20 (number of amino acids). The model applies one-hot encoding on every character, resulting in a binary vector of size *m*. It then applies a linear transformation to each vector to create a “character-level” embedding. These embeddings are concatenated and fed to a fully-connected layer, creating a “sequence-level” embedding. The final output layer is a linear layer with size equal to the number of prediction tasks *k*, followed by *k* task-dependent activation functions. In our case, *k* = 9, each task corresponds to an antibody in our train data, while our output activation functions are all sigmoids. This means our output will be 9 probabilities each corresponding to an escape probability of a different antibody.

When we refer to a model as a “single-task model” it means that *k* = 1, when *k* > 1 we refer to the model as a “multi-task model”. This means, that when comparing between multi-task and single task, we will have *k* single-task model, each corresponding to one antibody, while having a single multi-task model with *k* outputs. For training and evaluation we randomly split the data into 30% test and 70% train, run the model 100 times and report the performance distribution obtained using box-plots. Performance is measured in the binary case using the area under the ROC curve and in the continuous case using Pearson’s correlation between predictions and true value in test set. We use the Adam optimizer Kingma and Ba (2015) with a learning rate of 1e-4 and a maximum of 100 epochs. Our model loss function is the sum of all the tasks’ loss functions, where for each task we use the cross entropy loss function.

### 3.3 Training using a fixed embedding

Utilizing our multi-task model’s last hidden layer as a latent representation of mutations, we can predict other RBD properties such as binding. To this end, we add a linear layer after the embedding layer whose weights are trained using linear regression. When calculating escape probabilities, we use the sigmoid activation function in our output layer, so the training with fixed embedding is done *via* logistic regression (more precisely, linear regression on the inverse sigmoid of the escape data), meaning the task is identical to binding regression.

### 3.4 Data and materials availability

Code and data are available at https://github.com/bgmoshe/multi_tasking_antibodies.

## 4 Discussion

In this paper we develop a computational framework that harnesses systematic mutation screens in the receptor binding domain of the viral Spike protein for escape prediction. Unlike (Bozdaganyan et al., 2022) and (Greaney et al., 2021a), who demonstrate an approach to quantify binding to antibodies, we do not assume a predefined relation between the effect of different mutations, allowing us to have a more general model that is learned automatically from data. Furthermore, in contrast to (Hie et al., 2021) we can quantify mutation escape potential with respect to each antibody. Our framework allows us to infer a latent representation of mutations that preserves escape information. This is particularly useful for predictions regarding yet unseen antibodies or variants.

**FIGURE 4:**
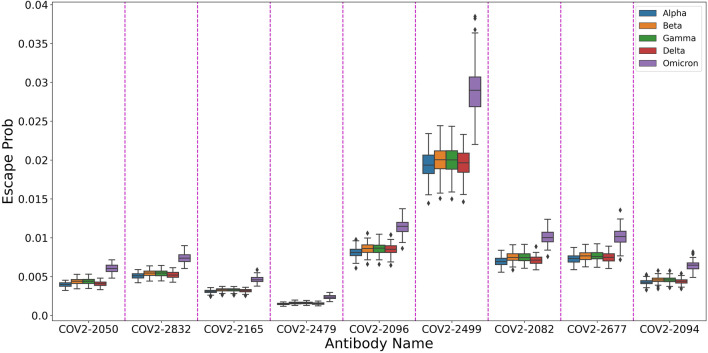
Predicted escape from antibodies for different SARS-CoV-2 variants.

In order to showcase this attribute, We used our trained model to predict the escape probabilities of variants of concern (as defined by the World Health Organization) as shown in Figure 4. The figure highlights the result of Planas et al. (2021) that Omicron has higher probability of evading antibodies than previous variants. We suggest that using our multi-task model one can provide information on the effect of multiple mutations at different sites, thus allowing researchers to focus on more likely variants of concern.

## Data Availability

The original contributions presented in the study are included in the article/Supplementary material, further inquiries can be directed to the corresponding author.
